# Microstructural Parameters of Bone Evaluated Using HR-pQCT Correlate with the DXA-Derived Cortical Index and the Trabecular Bone Score in a Cohort of Randomly Selected Premenopausal Women

**DOI:** 10.1371/journal.pone.0088946

**Published:** 2014-02-13

**Authors:** Albrecht W. Popp, Helene Buffat, Ursula Eberli, Kurt Lippuner, Manuela Ernst, R. Geoff Richards, Vincent A. Stadelmann, Markus Windolf

**Affiliations:** 1 Department of Osteoporosis, Inselspital, Berne University Hospital and University of Berne, Berne, Switzerland; 2 AO Research Institute Davos, Davos, Switzerland; Faculté de médecine de Nantes, France

## Abstract

***Background*:**

Areal bone mineral density is predictive for fracture risk. Microstructural bone parameters evaluated at the appendicular skeleton by high-resolution peripheral quantitative computed tomography (HR-pQCT) display differences between healthy patients and fracture patients. With the simple geometry of the cortex at the distal tibial diaphysis, a cortical index of the tibia combining material and mechanical properties correlated highly with bone strength ex vivo. The trabecular bone score derived from the scan of the lumbar spine by dual-energy X-ray absorptiometry (DXA) correlated ex vivo with the micro architectural parameters. It is unknown if these microstructural correlations could be made in healthy premenopausal women.

***Methods*:**

Randomly selected women between 20–40 years of age were examined by DXA and HR-pQCT at the standard regions of interest and at customized sub regions to focus on cortical and trabecular parameters of strength separately. For cortical strength, at the distal tibia the volumetric cortical index was calculated directly from HR-pQCT and the areal cortical index was derived from the DXA scan using a Canny threshold-based tool. For trabecular strength, the trabecular bone score was calculated based on the DXA scan of the lumbar spine and was compared with the corresponding parameters derived from the HR-pQCT measurements at radius and tibia.

***Results*:**

Seventy-two healthy women were included (average age 33.8 years, average BMI 23.2 kg/m^2^). The areal cortical index correlated highly with the volumetric cortical index at the distal tibia (R  =  0.798). The trabecular bone score correlated moderately with the microstructural parameters of the trabecular bone.

***Conclusion*:**

This study in randomly selected premenopausal women demonstrated that microstructural parameters of the bone evaluated by HR-pQCT correlated with the DXA derived parameters of skeletal regions containing predominantly cortical or cancellous bone. Whether these indexes are suitable for better predictions of the fracture risk deserves further investigation.

## Introduction

In addition to low bone mass, osteoporosis was characterized in the WHO Technical Report of 1994 [1] by the micro architectural deterioration of bone tissue. Predominantly driven by sex steroid hormone levels changing with aging, the structures of the long bones are altered by enhanced endosteal resorption and are only in part compensated by periosteal apposition [2]. This change translates into altered micro architecture of the trabecular and cortical bone in vivo as assessed by high resolution imaging techniques, such as high-resolution peripheral quantitative computed tomography (HR-pQCT) [3]. For the cortex, trabecularization of the cortical bone with aging has been suggested [4]. Accordingly, ex-vivo micrographs of the femoral cortex demonstrated increasing porosity and thinning of the cortex by coalescence of the intracortical pores [5].

Different studies have demonstrated the direct relationship between the mechanical properties of specific anatomical regions and their architecture ex vivo [6], [7] and in silico [8]–[10]. Even if the differences in the areal bone mineral density (aBMD) measurements by dual-energy X-ray absorptiometry (DXA) at the standardized sites were inexistent or mild, these structural parameters could distinguish between individuals with or without prevalent low velocity fractures [11], [12]. The standard regions of interest (ROI) used in the clinically applied HR-pQCT measurements at the distal radius and tibia were formerly focused on the trabecular bone [13], but meanwhile the quantification of the cortical bone derived indices, including the cortical porosity (Ct.Po) based on automated segmentation from HR-pQCT images, has been implemented [14].

A low aBMD is predictive of the individual fracture risk in postmenopausal women when measured at the hip, lumbar spine, or radius [15], [16]. The best predictive ability was observed when the measurements were performed at the same skeletal sites that sustained a fracture later. Whereas the tibial epiphysis (T-EPI) consists predominantly of trabecular bone, the tibial diaphysis (T-DIA) is predominantly composed of cortical bone. The authors previously showed that, in a cohort of randomly selected postmenopausal women, aBMD measured by DXA at both tibial subregions was predictive for non-vertebral fracture risk [17]. T-scores were generally higher at the T-DIA than at the T-EPI suggesting that the T-DIA might be suitable for identifying other contributors of an individual’s fracture risk, including parameters related to the shape and size.

Because of the growing share of cortex from distal to proximal the ratio of trabecular and cortical bone is highly variable at the tibial metaphysis [18]. A more proximal region of interest with thicker and more homogeneous cortices, such as the T-DIA, has a simple geometric structure, similar to a hollow cylinder. The combination of the aBMD at the T-DIA and the structural parameters derived from DXA scans and confirmed by HR-pQCT can improve the prediction of local bone strength ex vivo [19]. It is unknown whether this combination of material and structural competence can be confirmed by both of the techniques in a standardized procedure in vivo.

The trabecular bone score (TBS) is a DXA-derived parameter that focuses on the cancellous architecture. This score quantifies the gray-level texture out of the DXA scan of the lumbar spine [20]. TBS was shown not to correlate or to correlate weakly with the aBMD in postmenopausal women ranging from 0.2 to 0.58 [21]–[23]. In epidemiological studies, TBS was significantly lower in the women with incidental fractures compared to the controls [24]. Even improvements in the TBS could be observed in postmenopausal women under antiresorptive treatment in a randomized controlled trial [22]. While the correlation of this texture parameter to the parameters of microarchitecture, e.g., the degree of connectivity, the trabecular number and separation, was performed ex vivo by micro-computed tomography [25], little is known if these correlations can be confirmed by the HR-pQCT measurement of these parameters at the appendicular skeleton in vivo.

The aim of this study was to evaluate whether and to what extent the DXA-derived cortical and trabecular structural parameters correlate with the bone microstructural parameters assessed using HR-pQCT in premenopausal women.

## Materials and Methods

### Study participants

**Figure 1:**
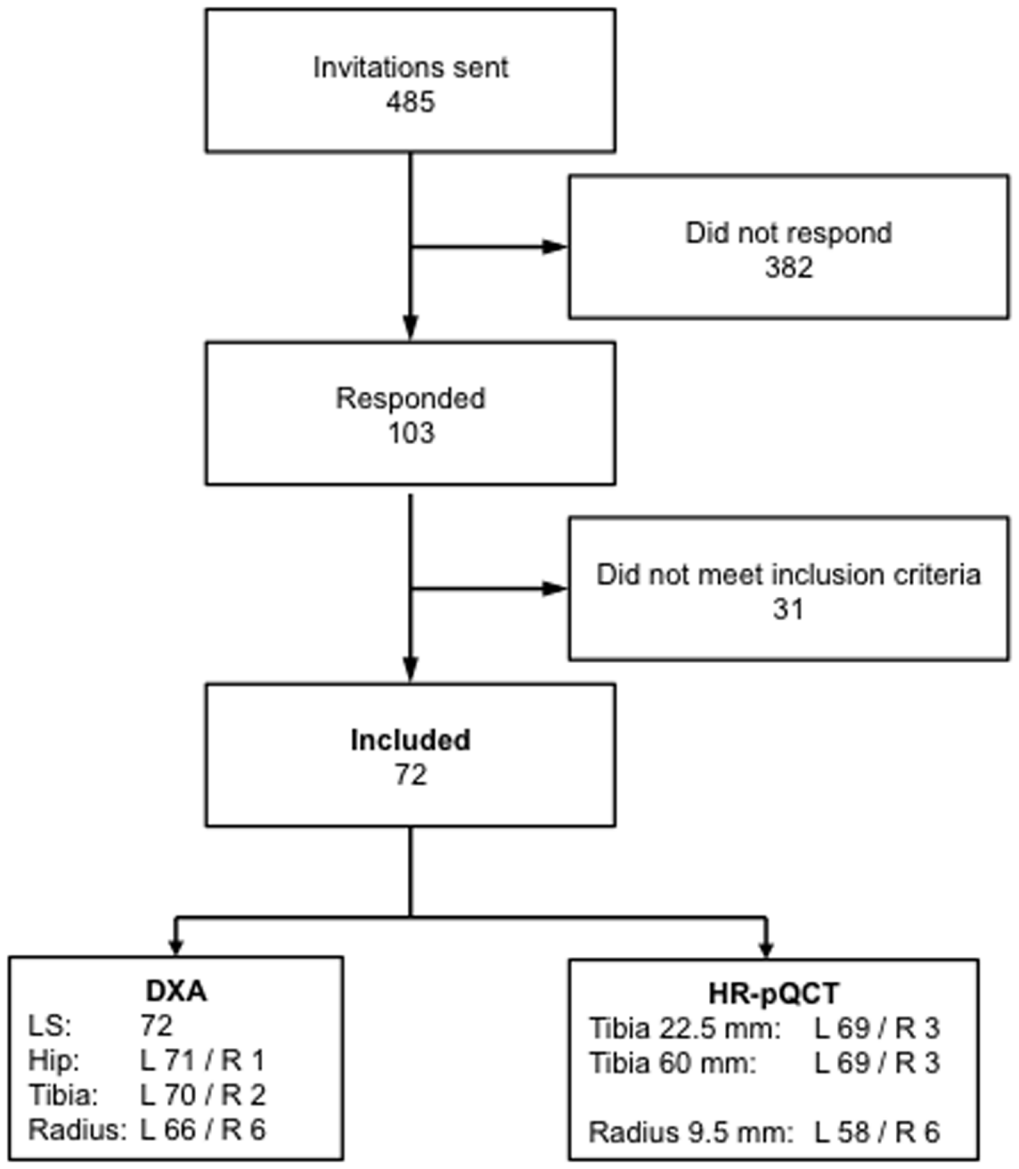
Consort flow diagram.

In December 2012, study invitations were mailed to a total of 485 women selected from commercially available address listings categorized by sex, age, and the region of Davos, Switzerland. Mobile, non-institutionalized, not pregnant (confirmed by a negative pregnancy test) randomly selected women between 20–40 years old were eligible. The exclusion criteria were a known bone disease or immobilization (relative or absolute immobilization was defined as unable to stand or walk at least one hour per day). In total, 72 women were included in this study, sixty-nine of these women were white. The left upper and lower extremities were examined or the nonfractured contralateral extremity in cases of prior fracture on the left side (Fig. 1). All of the measurements occurred at the AO Research Institute Davos, Switzerland.

### Ethical Statement

The study was approved by the local ethics committee for medical sciences (Ethics Committee of the Canton of Zurich, Switzerland, registration number 2012-0464). All of the participants provided prior written informed consent and were monetarily compensated for their efforts.

### DXA Measurements

**Figure 2:**
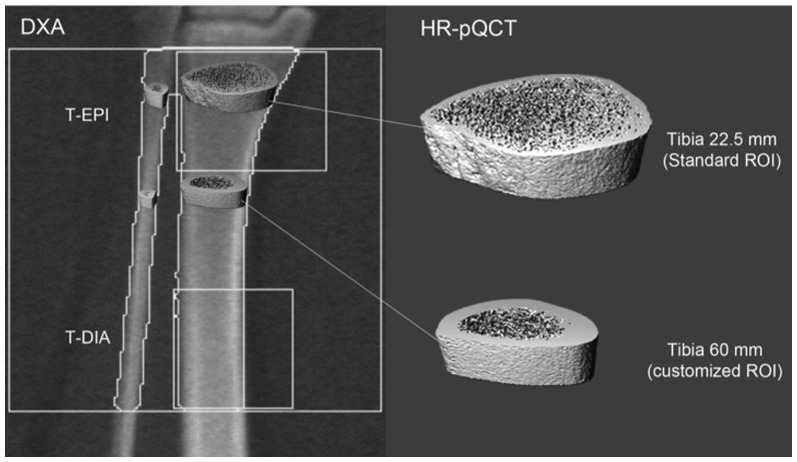
Regions of interest at the left distal tibia by DXA (left) and the volumes of interest as 3D reconstructions by HR-pQCT (right).

DXA densitometer measurements (Hologic Discovery C™, Hologic, Bedford, MA, USA) were performed with a mobile device provided by the Department of Osteoporosis, University Hospital Berne, Switzerland. aBMD was measured at the lumbar spine (L1-4; LS), the femoral neck (FN) and total hip (TH), the distal radius (Rad 1/3), and at the two customized subregions of the distal tibia (T-EPI, T-DIA). The detailed standardized procedures for the tibial BMD measurements by DXA were previously published [26]. The ROI is defined as the 120 mm height and 129 mm width area that starts 10 mm above the top of the ankle joint space. T-EPI corresponds to the distal 40 mm of the ROI, and T-DIA corresponds to the proximal 40 mm of the ROI (Fig. 2). aBMD is expressed in grams of hydroxyapatite (HA) per square centimeter of the projected area. The obtained aBMD values of the femoral neck were expressed as the standard deviation (SD) of the mean value of the young healthy population (T-scores) derived from the National Health and Nutrition Examination Survey in the United States of America (NHANES III) [27]. The scans were performed according to the manufacturer’s guidelines, and the analyses were conducted according to the recommendations of the International Society of Clinical Densitometry. Spine phantom scanning was routinely performed on a daily basis for quality control. A least significant change at the 95% confidence level of 0.020 g/cm^2^ for the aBMD at the total hip was achieved as the reproducibility index for the technician involved.

### DXA-Derived Parameters of Cortical and Trabecular Bone

**Figure 3:**
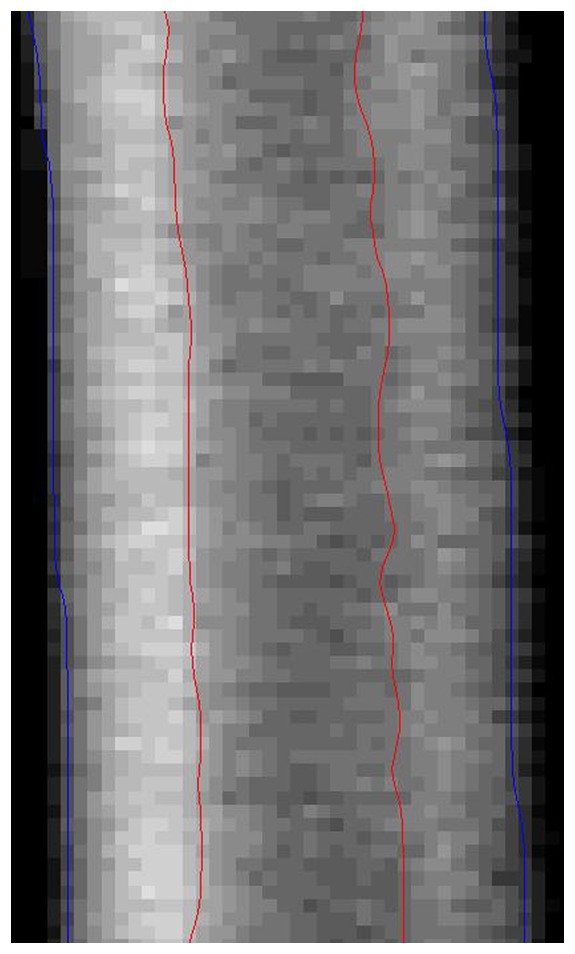
ROI of a representative DXA data set of the tibial diaphysis. The cortex edges as derived from image filtering are depicted as blue (outer diameter) and red lines (intramedullary channel).



Assuming a hollow cylindrical shape of the T-DIA, the DXA-derived cortical thickness (Ct.Th) was calculated using the DICOM images of the DXA scan at the distal tibia. DXA gray-scale images were imported into Matlab™ programming environment (MATLAB™, MathWorks Inc., Natick, MA, USA). The region of interest was automatically extracted for all data sets. Image data was segmented using an edge detection filter based on the algorithm proposed by Canny [28]. Canny threshold was kept constant at 0.05 resulting in a binary image showing the inner and outer borders of the cortex. A Butterworth filter was applied for smoothing the edges (Fig. 3). Cortex diameter (DIA) was defined as mean pixel distance between the outermost edges. Ct.Th was calculated as mean distance between outer and inner edges of the medial cortex projection, because the individual variation of Ct.Th is wider at the medial cortex than at the lateral cortex, i.e., the cortex closer to the fibula (own observation, unpublished data). Values were transformed to millimeter by isometric scaling (2.0 pixels/mm). Estimating the bony cross section as ideal tube the polar moment of inertia (pMOI) was computed according to formula I.

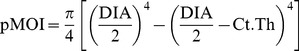
(I)A new parameter was introduced as cortical index for diaphyseal bone strength (CI), defined as product of a material (aBMD) and a structural component (pMOI). Because this CI is calculated from the two-dimensional DXA data, it is called the areal CI (aCI) in formula II. 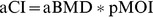
(II)


TBS was evaluated in the identical ROI as those used for the LS BMD. The dimensionless TBS values were calculated as the mean of the individual measurements for vertebrae L1-L4 from the DXA using TBS iNsight® Software (version 1.8.2; Med-Imaps, Bordeaux, France).

### HR-pQCT Measurements

HR-pQCT (XtremeCT, Scanco Medical AG, Brüttisellen, Switzerland) measurements were performed by immobilizing the left forearm and the left distal tibia in a carbon fiber shell and scanning as previously described [29]–[31]. In addition to the standard ROI at the radius (radius 9.5 mm) and at the tibia (tibia 22.5 mm), a third customized ROI was scanned starting at the distal tibia 60 mm above the endplate (tibia 60 mm) to increase the amount of cortex measured (Fig. 2). A stack of 110 parallel CT slices was acquired at the distal starting points of all of the sites in the proximal direction using 60 kVp and a 126 mm field of view reconstructed across an image matrix size of 1536×1536, with a voxel size of 82 µm. These provided a 3D image of 9.02 mm in the axial direction. The attenuation data were converted to the equivalent hydroxyapatite (HA) densities. The manufacturer’s phantom was scanned daily for quality control.

The bone morphological parameters were evaluated using the standard protocols provided by the manufacturer. The parameters of interest were the total volumetric bone mineral density (vBMD), the bone volume fraction (BV/TV), the trabecular bone density (Tb. BMD), the trabecular thickness (Tb.Th), the trabecular number (Tb.N), the trabecular separation (Tb.Sp), the inhomogeneity of the trabecular network, defined as Tb.1/N.SD, and connectivity density of the trabecular network (Conn.D), the cortical bone density (Ct.BMD) and the cortical thickness (Ct.Th). The cortical porosity (Ct.Po) and the polar moment of inertia (pMOI), as a structural index of resistance to torsion, were calculated using an automated image processing chain [14], [32]. As an analogue to the calculation from DXA, the vBMD at both ROI of the tibia multiplied by the pMOI leads to the volumetric cortical index (vCI) as a potential parameter of cortical strength. Scans with severe movement artifacts, such as a blurry trabecular area or skewed or discontinued cortical faces, were excluded. Based on the categories (1 = best, 5  =  worst), seven forearm scans of grade 3 and higher were excluded [33]. Additionally, a single forearm measurement by HR-pQCT was lost for technical reasons.

### Statistical Analysis

The analyses were conducted with PASW statistical software (version 21.0; SPSS™, Chicago, IL). The two-sided p values <0.05 were considered to indicate statistical significance. The descriptive data were presented as the mean ± standard deviation (SD). After checking the variables of interest for normality (Kolmogorov-Smirnov test), the correlations between the structural parameters were expressed as R Pearson’s coefficient. To compare correlations, Fisher’s transformation was applied.

## Results

### Baseline Characteristics

Seventy-two healthy premenopausal women were included in the study. The anthropometric baseline characteristics are shown in Table 1. The mean age was 33.8 years; the mean height and BMI were 167.6 cm and 23.2 kg/m^2^, respectively. Forty-two women were nulliparas, and 9 of 72 women were current smokers. The mean calcium intake was 816 mg per day. Thirty-eight females had prevalent fractures, mainly in the forearms (18), phalanges (14) and ankle (6). Twenty-two women or 30.6 % reported a potentially positive family history for osteoporosis, i.e. any self-reported major osteoporotic fracture (clinical spine, forearm, hip or shoulder fracture) among first-/second-grade relatives. As shown in Table 2, the femoral neck BMD was 0.853 g/cm^2^, corresponding to a mean NHANES T-score of 0.04 (±1.08) SD. All of the areal and volumetric density parameters at all of the ROI were normally distributed (Tables 2 and 3). While the magnitude of extent of the vBMD was similar at the standard ROI of the radius 9.5 mm and the tibia 22.5 mm, the one at the tibia 60 mm was higher because of the higher portion of the cortical compartment. The Ct.Po was lower at the tibia 60 mm compared to the tibia 22.5 mm (1.2±0.5 % vs. 4.3±1.2 %).

**Table 1:** Baseline characteristics.

n = 72	mean	SD
Age (years)	33.8	5.5
Height (cm)	167.6	6.2
Weight (kg)	65.2	12.1
BMI (kg/m2)	23.2	4.1
Calcium intake (mg per day)	816.0	402
	n	%
Nulliparas	42	58.3
Smoker total	19	26.4
former	10	13.9
current	9	12.5
Family history for osteoporosis	22	30.6
BMI < 18.5 kg/m^2^	3	4.2
Ever glucocorticoid use	6	8.3

**Table 2:** Areal BMD and the structural derivates by DXA.

DXA		mean	SD
**aBMD**			
FN	g/cm^2^	0.853	0.119
TH	g/cm^2^	0.952	0.116
LS	g/cm^2^	1.039	0.138
Rad 1/3	g/cm^2^	0.712	0.046
T-DIA	g/cm^2^	1.362	0.108
T-EPI	g/cm^2^	0.750	0.096
**Structural derivates**			
Ct.Th (T-DIA)	mm	3.8	0.58
pMOI (T-DIA)	mm^4^	1.9298	0.64
aCI	g*cm^2^	2.62	0.856
TBS	-	1.399	0.087

**Table 3:** Volumetric BMD and the structural parameters by HR-pQCT.

HR-pQCT							
		radius 9.5 mm		tibia 22.5 mm		tibia 60 mm	
	*Unit*	*mean*	*SD*	*mean*	*SD*	*mean*	*SD*
total vBMD	mgHA/cm^3^	307.6	53.3	303.2	50.1	550.3	79.3
BV/TV	%	13.7	2.8	14.1	2.9	7.6	2.6
**Cortical parameters**							
Ct.BMD	mgHA/cm^3^	846.2	44.7	874.8	33.2	997.9	20.9
Ct.Th	mm	0.760	0.151	1.229	0.221	2.521	0.267
pMOI	mm^4^	7694	2228	41104	11140	20031	4967
Ct.Po	%	2.8	1.0	4.3	1.2	1.2	0.5
vCI	gHA*cm	0.234	0.072	1.231	0.352	1.079	0.222
**Trabecular parameters**							
Tb.BMD	mgHA/cm^3^	164.4	34.2	169.7	34.5	91.2	31.2
Tb.N	1/mm	1.944	0.246	1.912	0.312	1.476	0.424
Tb.Th	mm	0.070	0.009	0.074	0.011	0.052	0.011
Tb.Sp	mm	0.453	0.073	0.464	0.094	0.690	0.247
Inhomogeneity	mm	0.187	0.038	0.209	0.053	0.373	0.177
Conn.D	mm^−3^	3.300	0.816	3.39	1.01	1.020	0.570

### Correlation Analysis Between the Cortical Parameters

**Figure 4:**
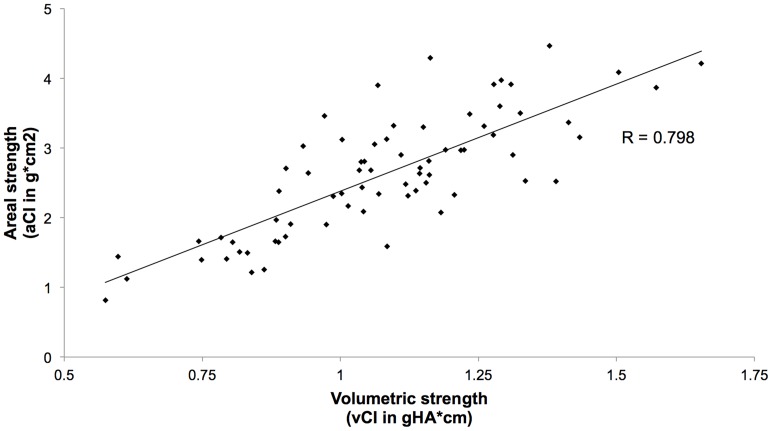
Correlation between the aCI (DXA) and the vCI (HR-pQCT) at the distal tibia.

For the cortex at the distal tibia, a significant correlation (R  =  0.74, p<0.01, Table 4) was found between the CI measured at the tibia 22.5 mm by DXA and HR-pQCT, indicating that the bone strengths assessed by both techniques were predictive of each other. The correlation coefficient was even higher at the tibia 60 mm (Fig. 4), where the portion of cortical bone is more substantial, but without reaching statistical significance (p = 0.3).

**Table 4:** Correlations between the parameters derived from DXA and HR-pQCT.

	DXA									
HR-pQCT radius tibia 22.5 mm tibia 60 mm	aBMD FN	aBMD TH	aBMD LS	aBMD Rad 1/3	aBMD T-DIA	aBMD T-EPI	Ct.Th T-DIA	pMOI T-DIA	aCI T-DIA	TBS
total vBMD	**0.501 0.532** *0.295*	**0.586 0.603 0.307**	**0.334** *0.275* 0.020	**0.521 0.496 0.429**	**0.459 0.570 0.601**	**0.564 0.748 0.467**	*0.259* **0.586 0.566**	–**0.440** –**0.333** –**0.600**	–*0.337* –0.177 –**0.433**	0.242 *0.254* 0.029
BV/TV	**0.544 0.659 0.594**	**0.707 0.736 0.644**	**0.608 0.631 0.680**	**0.363 0.407** 0.227	**0.457 0.558 0.442**	**0.736 0.889 0.664**	**0.374 0.521** *0.247*	–0.117 0.075 0.169	0.015 0.220 *0.290*	**0.494 0.508 0.572**
Ct.BMD	*0.300* 0.196 –0.101	*0.288* 0.210 –0.113	0.045 –0.117 –*0.293*	**0.465 0.377** *0.257*	0.241 0.183 0.073	0.209 0.184 –0.068	0.065 0.119 –0.007	–**0.492** –**0.436** –**0.525**	–**0.417** –**0.370** –**0.494**	0.008 –0.017 –*0.295*
Ct.Th	**0.441 0.399 0.382**	**0.506 0.492 0.440**	*0.288* 0.163 0.186	**0.525 0.446 0.471**	**0.422 0.542 0.791**	**0.506 0.650 0.665**	0.230 **0.612 0.755**	–**0.326** –*0.285* –*0.242*	–0.212 –0.134 –0.029	0.154 0.185 0.167
pMOI	0.115 0.205 0.149	*0.295* **0.302** *0.234*	**0.479 0.514 0.412**	–0.003 –0.030 –0.063	0.135 0.118 0.073	**0.448 0.368 0.466**	0.245 0.105 –0.002	**0.704 0.829 0.892**	**0.748 0.821 0.897**	*0.276* **0.351 0.315**
vCI	**0.411 0.542 0.452**	**0.640 0.680 0.564**	**0.684 0.708 0.588**	**0.324** *0.284 0.267*	**0.408 0.477 0.585**	**0.764 0.820 0.714**	**0.384 0.461 0.450**	**0.417 0.599 0.645**	**0.542 0.729 0.798**	**0.408 0.512 0.447**
Ct.Po	0.016 0.135 0.271	0.109 0.200 0.259	0.289 **0.415** *0.298*	–0.166 –0.122 –0.024	0.128 0.208 *0.301*	0.236 **0.308** *0.300*	0.110 *0.252* **0.308**	0.171 **0.338** 0.131	0.196 **0.392** 0.208	0.227 0.155 *0.247*
Tb.BMD	**0.544 0.659 0.593**	**0.707 0.735 0.643**	**0.608 0.630 0.681**	**0.363 0.408** 0.227	**0.458 0.559 0.442**	**0.737 0.889 0.665**	**0.373 0.522** *0.248*	–0.118 0.073 0.167	0.014 0.221 *0.288*	**0.493 0.508 0.570**
Tb.N	**0.467 0.525 0.604**	**0.532 0.565 0.611**	**0.517 0.645 0.565**	*0.307 0.268 0.274*	**0.400 0.420 0.552**	**0.589 0.627 0.636**	0.230 *0.260* **0.321**	–0.058 *0.253* 0.189	0.056 **0.358 0.330**	**0.432 0.527 0.497**
Tb.Th	**0.432 0.353** 0.115	**0.624 0.411** 0.167	**0.455** 0.136 *0.287*	*0.296 0.280* –0.082	**0.363** *0.293* –0.098	**0.608 0.533** 0.167	**0.389 0.437** –0.035	–0.152 –0.195 –0.002	–0.046 –0.011 –0.015	**0.373** 0.109 0.188
Tb.Sp	–**0.452** –**0.517** –**0.546**	–**0.551** –**0.570** –**0.582**	–**0.528** –**0.672** –**0.592**	–**0.322** –*0.280* –*0.301*	–**0.415** –**0.422** –**0.526**	–**0.614** –**0.656** –**0.610**	–*0.272* –*0.287* –*0.295*	0.079 –*0.257* –0.208	––0.040 **-0.360** –**0.337**	–**0.428** –**0.572** –**0.570**
Inhomogeneity	–**0.475** –**0.482** –**0.571**	–**0.531** –**0.503** –**0.584**	–**0.487** –**0.634** –**0.536**	–*0.306* –*0.233* –**0.355**	–*0.394* –*0.322* –**0.550**	–**0.547** –**0.563** –**0.595**	–0.222 –0.183 –**0.306**	0.071 –*0.264* –0.100	–0.043 –**0.344** –*0.243*	–**0.408** –**0.498** –**0.510**
Conn.D	**0.425 0.432 0.466**	**0.496 0.464 0.473**	**0.499 0.596 0.575**	0.188 0.142 0.092	**0.377 0.346** *0.279*	**0.558 0.497 0.449**	0.228 0.155 0.040	0.061 **0.310 0.376**	0.172 **0.397 0.450**	**0.416 0.461 0.428**

p<0.05 (italic); **p<0.01 (bold).**

### Correlation Analysis Between the DXA-derived and HR-pQCT Parameters of the Trabecular Bone

The TBS of L1-4 correlated significantly (p<0.01) with the following parameters at the radius 9.5 mm and the tibia 22.5 mm: positively with the Tb.BMD, BV/TV, Tb.N, Conn.D, and negatively with the TbSp, inhomogeneity (Table 4). Correlations between the TBS and the Tb.Th, the total vBMD, the Ct.BMD, the Ct.Th or the Ct.Po were absent or inconsistent between the different skeletal sites. Among these healthy women, the TBS correlated with the aBMD at the lumbar spine and femoral neck (R = 0.660 and 0.532).

## Discussion

The CI and TBS represent DXA derived parameters of the cortical and cancellous bone. This study showed for the first time that the corresponding structural parameters of the bone evaluated by HR-pQCT correlate with these DXA derived parameters in a cohort of randomly selected premenopausal women. In clinical practice, the aBMD is the only bone-specific parameter currently used to assess the fracture risk. By integrating the cortical and cancellous bone of a defined skeletal area, DXA scans provide bone mass of the projected area as expressed as the aBMD. If specific skeletal sites revealed a substantial amount of cortical or cancellous bone, these structural parameters could be quantified. The distal tibial diaphysis offers simple geometry similar to a hollow cylinder and a high percentage of cortical bone. Ex vivo material and structural characteristics of the bone at the T-DIA were previously shown to be predictive of the bone strength assessed by biomechanical testing [19]. As shown in the present study, the determination of the pMOI on a DXA scan of the tibia allowed for calculation of the CI by integrating the potential bone material (BMD) and the mechanical properties (pMOI). This index correlated highly with the index obtained by applying HR-pQCT. The correlation between those cortical parameters is stepwise, increasing from the radius 9 mm to the tibia 22.5 mm to the tibia 60 mm, which may reflect that a higher cortex portion better predicts its quantity, even if it is enhanced by considering two subregions of the same tibia. Interestingly, the cortical porosity at the distal tibia decreases the more proximal it is measured, which might reflect less transition from trabecular to cortical bone in the tibial diaphysis. Further studies are needed to confirm if this standardized quantification of the cortex in individuals at risk is predictive for the incidences of fractures.

By focusing on the cancellous bone contained in the lumbar spine, this ROI is predestinated for the assessment of the trabecular structure. TBS is a texture parameter that evaluates the pixel gray-level variations in DXA images of the lumbar spine. Ex vivo, these variations reflect the altered micro architecture of the trabecular bone such as the Conn.D, Tb.Sp or Tb.N assessed by high-resolution micro computed tomography [20], [25]. In our cohort of healthy young women, the TBS correlated highly with these parameters of the microstructures, although the resolution of HR-pQCT is lower compared to that used ex vivo. Similar correlations were described recently in a pooled cohort of pre- and postmenopausal Chinese American and white women with exception that the strongest association has been found between TBS and vBMD [34]. In postmenopausal women with osteoporosis, the TBS did not correlate or correlated weakly with the aBMD of the lumbar spine [22]. In contrast to that, R was highly significant between aBMD at the LS and TBS in this study in healthy controls indicating that normal bone mass and not altered, homogeneous texture correlate highly with each other.

This study has some limitations. All of the measurements were performed in healthy premenopausal women and will not necessarily translate into findings of other cohorts, e.g., postmenopausal women or men. The sample might be selective (e.g., the subjects were from rural mountain areas; the regional calcium rich dietary habits of the subjects may have had an effect). For both of the bone structures, only one skeletal region was considered, and the findings may differ when applying a similar methodology to other sites of the skeleton. Whether the CI reflects more than regional bone strength is speculative. The strengths of the study included the number of randomly selected healthy women and the consistency of the study results of the hip aBMD to the data obtained by the NHANES III, which demonstrates the balance of the sample.

In this cohort of randomly selected healthy women, microstructural parameters of bone assessed by the 3D technique were predictable through information deducted from regular 2D DXA scans. This approach may add information to improve the prediction of individual fracture risks in a clinical setting.
