# Correction: Microstructural Parameters of Bone Evaluated Using HR-pQCT Correlate with the DXA-Derived Cortical Index and the Trabecular Bone Score in a Cohort of Randomly Selected Premenopausal Women

**DOI:** 10.1371/journal.pone.0098167

**Published:** 2014-05-16

**Authors:** The PLOS ONE Staff

There is an error in Table 4. The columns and rows were incorrectly formatted. *PLOS ONE* apologizes for the error. You can view the corrected table here.

**Table 4:** Correlations between the parameters derived from DXA and HR-pQCT.

	DXA									
HR-pQCT radius tibia 22.5 mm tibia 60 mm	aBMD FN	aBMD TH	aBMD LS	aBMD Rad 1/3	aBMD T-DIA	aBMD T-EPI	Ct.Th T-DIA	pMOI T-DIA	aCI T-DIA	TBS
total vBMD	**0.501 0.532** *0.295*	**0.586 0.603 0.307**	**0.334** *0.275* 0.020	**0.521 0.496 0.429**	**0.459 0.570 0.601**	**0.564 0.748 0.467**	*0.259* **0.586 0.566**	**−0.440 −0.333 −0.600**	*−0.337* −0.177 **−0.433**	0.242 *0.254* 0.029
BV/TV	**0.544 0.659 0.594**	**0.707 0.736 0.644**	**0.608 0.631 0.680**	**0.363 0.407** 0.227	**0.457 0.558 0.442**	**0.736 0.889 0.664**	**0.374 0.521** *0.247*	−0.117 0.075 0.169	0.015 0.220 *0.290*	**0.494 0.508 0.572**
Ct.BMD	*0.300* 0.196 −0.101	*0.288* 0.210 −0.113	0.045 −0.117 *−0.293*	**0.465 0.377** *0.257*	0.241 0.183 0.073	0.209 0.184 −0.068	0.065 0.119 −0.007	**−0.492 −0.436 −0.525**	**−0.417 −0.370 −0.494**	0.008 −0.017 *−0.295*
Ct.Th	**0.441 0.399 0.382**	**0.506 0.492 0.440**	*0.288* 0.163 0.186	**0.525 0.446 0.471**	**0.422 0.542 0.791**	**0.506 0.650 0.665**	0.230 **0.612 0.755**	**−0.326** *−0.285 −0.242*	−0.212 −0.134 −0.029	0.154 0.185 0.167
pMOI	0.115 0.205 0.149	*0.295* **0.302** *0.234*	**0.479 0.514 0.412**	−0.003 −0.030 −0.063	0.135 0.118 0.073	**0.448 0.368 0.466**	0.245 0.105 −0.002	**0.704 0.829 0.892**	**0.748 0.821 0.897**	*0.276* **0.351 0.315**
vCI	**0.411 0.542 0.452**	**0.640 0.680 0.564**	**0.684 0.708 0.588**	**0.324** *0.284 0.267*	**0.408 0.477 0.585**	**0.764 0.820 0.714**	**0.384 0.461 0.450**	**0.417 0.599 0.645**	**0.542 0.729 0.798**	**0.408 0.512 0.447**
Ct.Po	0.016 0.135 0.271	0.109 0.200 0.259	0.289 **0.415** *0.298*	−0.166 −0.122 −0.024	0.128 0.208 *0.301*	0.236 **0.308** *0.300*	0.110 *0.252* **0.308**	0.171 **0.338** 0.131	0.196 **0.392** 0.208	0.227 0.155 *0.247*
Tb.BMD	**0.544 0.659 0.593**	**0.707 0.735 0.643**	**0.608 0.630 0.681**	**0.363 0.408** 0.227	**0.458 0.559 0.442**	**0.737 0.889 0.665**	**0.373 0.522** *0.248*	−0.118 0.073 0.167	0.014 0.221 *0.288*	**0.493 0.508 0.570**
Tb.N	**0.467 0.525 0.604**	**0.532 0.565 0.611**	**0.517 0.645 0.565**	*0.307 0.268 0.274*	**0.400 0.420 0.552**	**0.589 0.627 0.636**	0.230 *0.260* **0.321**	−0.058 *0.253* 0.189	0.056 **0.358 0.330**	**0.432 0.527 0.497**
Tb.Th	**0.432 0.353** 0.115	**0.624 0.411** 0.167	**0.455** 0.136 *0.287*	*0.296 0.280* −0.082	**0.363** *0.293* −0.098	**0.608 0.533** 0.167	**0.389 0.437** −0.035	−0.152 −0.195 −0.002	−0.046 −0.011 −0.015	**0.373** 0.109 0.188
Tb.Sp	**−0.452 −0.517 −0.546**	**−0.551 −0.570 −0.582**	**−0.528 −0.672 −0.592**	**−0.322** *−0.280 −0.301*	**−0.415 −0.422 −0.526**	**−0.614 −0.656 −0.610**	*−0.272 −0.287 −0.295*	0.079 *−0.257* −0.208	−0.040 **−0.360 −0.337**	**−0.428 −0.572 −0.570**
Inhomogeneity	**−0.475 −0.482 −0.571**	**−0.531 −0.503 −0.584**	**−0.487 −0.634 −0.536**	*−0.306 −0.233* **−0.355**	*−0.394 −0.322* **−0.550**	**−0.547 −0.563 −0.595**	−0.222 −0.183 **−0.306**	0.071 *−0.264* −0.100	−0.043 **−0.344** *−0.243*	-**0.408 −0.498 −0.510**
Conn.D	**0.425 0.432 0.466**	**0.496 0.464 0.473**	**0.499 0.596 0.575**	0.188 0.142 0.092	**0.377 0.346** *0.279*	**0.558 0.497 0.449**	0.228 0.155 0.040	0.061 **0.310 0.376**	0.172 **0.397 0.450**	**0.416 0.461 0.428**

*p<0.05*; **p<0.01.**
